# A Cross-Sectional Analysis of Online Information Available to Prospective Facial Plastic and Reconstructive Surgery Fellowship Applicants

**DOI:** 10.7759/cureus.101669

**Published:** 2026-01-16

**Authors:** Natasha Mayer, Micah K Harris, Anthony Tang, Michael Calcaterra, Christina M Yver

**Affiliations:** 1 Otolaryngology - Head and Neck Surgery, University of Pittsburgh Medical Center, Pittsburgh, USA

**Keywords:** academic and private, facial plastics and reconstructive surgery, fellowship training, quality improvement, website evaluation

## Abstract

Background

Otolaryngology residents seeking training in facial plastic and reconstructive surgery (FPRS) often rely on the internet to assess potential fellowship programs. Despite this, the availability and reliability of content provided on FPRS websites have not been assessed.

Methodology

In this cross-sectional study, the 2023 American Academy of Facial Plastic and Reconstructive Surgery (AAFPRS) Handbook was used as a comprehensive registry of existing FPRS fellowship programs. All 65 listed programs (100%) were evaluated against 29 predefined criteria and compared with their associated program-created websites to assess the availability of applicant-relevant information. Descriptive analyses were used for categorical criteria, Fisher’s exact test was applied to compare website presence by program type, and Wilcoxon rank-sum testing was used for continuous measures of criteria reporting.

Results

Of the 65 FPRS fellowship programs listed, 43 (66.2%) had an associated program-created website. Overall, program-created websites reported significantly fewer applicant-relevant criteria than the AAFPRS Handbook, fulfilling an average of 9.8 of 29 criteria (33.9%) compared with 15.6 of 29 criteria (53.8%), respectively (p < 0.05). The AAFPRS Handbook provided more consistent reporting of core program characteristics, but did not include information on current fellows, fellowship graduates, the number of fellowship positions, interview details, or program coordinator contact information. In contrast, program-created websites more frequently reported applicant-centered information, including current fellows (44.2%), fellowship graduate placement (23.3%), and the number of fellowship positions (29.3%). In stratified analyses, university-affiliated programs were significantly more likely to maintain a program-created website than private practice-based programs (75.5% vs. 25.0%, p < 0.05). Among programs with websites, private practice-based fellowships reported substantially fewer applicant-relevant criteria than academic-affiliated programs (16.1% vs. 35.2% of the total criteria fulfilled, p < 0.05).

Conclusions

FPRS fellowship websites and data from the AAFPRS lack many key criteria valued by prospective applicants. Private practice-based fellowships are notably underrepresented online, which may limit applicants’ ability to gather information about programs outside of the academic arena.

## Introduction

Facial plastic and reconstructive surgery (FPRS) is a competitive sub-specialty of otolaryngology. Since 2014, the number of FPRS fellowships nationwide has increased from 44 to 65 (32%), while applicants participating in the match process have increased from 50 in 2014 to 57 in 2023 (12%) [1]. With this rise in interest, it is important for prospective FPRS fellows to be able to comprehensively evaluate fellowship programs to ensure optimal fit. Applicants often rely on online information to evaluate programs. Therefore, the availability of reliable and relevant information through online resources is of significance.

A prior survey of FPRS fellows and applicants identified the type of practice, location, exposure to the business of medicine/practice management, exposure to surgical techniques, and postgraduate plans to be important factors [2]. Many prior studies have evaluated the availability of similar criteria on fellowship websites [3-9]. Within otolaryngology, fellowship websites from pediatric otolaryngology, rhinology/skull base surgery, neurotology, head and neck surgery, and laryngology have been assessed. Each of these analyses has shown that information provided by fellowship websites is lacking [3-6]. Notably, this type of systematic assessment has not previously been performed for FPRS fellowship programs, representing a critical gap in the literature that this study seeks to address.

There are two online resources from which applicants draw information: the American Academy of Facial Plastic and Reconstructive Surgery (AAFPRS) and program-created websites [7]. Thus, the goal of this study was to assess the availability and breadth of information available from these two sources. Relevant criteria were determined based on prior surveys of FPRS fellows as well as other surgical specialties. A cross-sectional analysis of these platforms may inform prospective FRPS applicants regarding which resources are most useful during the information-gathering phase of the fellowship application process.

This article was previously presented as a meeting abstract at the 2024 Combined Otolaryngology Spring Meeting (COSM) on May 16, 2024.

## Materials and methods

Website evaluation

A comprehensive list of FPRS fellowship programs was identified from the AAFPRS Handbook on July 10, 2024 [7]. Corresponding fellowship websites were identified using Google (Google Inc., Mountain View, CA, USA) as a search engine between July 2024 and January 2025. Institutions offering multiple fellowships (private practice vs. academic track) were matched based on their respective program directors as reported by the AAFPRS. Programs that offered department websites but lacked a fellowship-specific tab were excluded.

Criteria selection

The utility of each website was evaluated for 29 key criteria. These criteria were determined based on prior analyses of otolaryngology fellowship websites in addition to prior surveys of FPRS applicants and fellows regarding what they deem important in a fellowship. Prior analyses of otolaryngology fellowship websites for laryngology, rhinology/skull base surgery, head and neck, neurotology, and pediatric otolaryngology have reviewed factors such as operative volume, breadth of surgical exposure, research opportunities, location, along with criteria such as current fellows, post-fellowship placement, number of fellowship positions, call schedule, general program description, and contact information [3-6]. In addition to these criteria, we included criteria specific to FPRS, including exposure to Mohs surgery, aging face surgery, rhinoplasty, micro-reconstruction, gender-affirming surgery, and exposure to the business of medicine, types of experiences that were found to be highly valued by prospective FRPS fellows when surveyed [2]. We also included research opportunities, conference attendance, general case volume, detailed case descriptions, didactic learning, evaluation criteria, program description, program coordinator and director contact information, current fellows, description of fellows, teaching requirements, number of fellowship positions, affiliation with academic hospitals, interview information, fellowship graduates, faculty description, benefits, vacation/sick pay, salary, and information on the surrounding area.

All criteria were weighted equally and scored on a binary scale (0 or 1). Websites were evaluated by two independent reviewers (NM and MC), and any discrepancies in scoring were resolved with the input of a third independent reviewer (CMY). All accessed websites were publicly available, and as a result, this study was considered exempt from Institutional Review Board approval by the University of Pittsburgh.

Statistical analysis

Descriptive statistics were used to summarize the availability of reported criteria across program-created fellowship websites and the AAFPRS Handbook. Continuous variables, including the number of reported criteria per program and the percentage of total criteria fulfilled, were compared between the AAFPRS Handbook and program-created websites as well as between academic-affiliated and private practice-based fellowship programs using the Wilcoxon rank-sum test. Differences in the presence of program-created fellowship websites by program type (university-affiliated vs. private practice-based) were assessed using Fisher’s exact test, given cell sizes <5. All other comparisons were descriptive in nature. Statistical analyses were performed using Microsoft Excel (Microsoft Corp., Redmond, WA, USA) and Prism Version 10 (GraphPad, La Jolla, CA, USA).

## Results

Fellowship website characteristics

Of the 65 fellowship programs listed in the AAFPRS handbook, 43 (66.2%) had corresponding program-created fellowship websites. Program-created websites reported fewer criteria and demonstrated greater variability, fulfilling an average of 9.8 ± 11.3 of the 29 key criteria, compared with an average of 15.6 ± 1.9 criteria reported in the AAFPRS Handbook. In a descriptive analysis, the distribution of reported criteria across program-created websites and the AAFPRS Handbook is summarized in Table 1, with descriptions of each evaluation characteristic provided in Table 2.

**Table 1:** Descriptive comparison of content availability on facial plastic and reconstructive surgery fellowship websites versus the AAFPRS Handbook. Program-created websites are a subset of programs listed in the AAFPRS handbook and are therefore not independent. In this table, individual criteria are summarized descriptively by online resource type. Wilcoxon rank-sum testing was applied only to the total number of criteria reported per program and is reported in the text. AAFPRS = American Academy of Facial Plastics and Reconstructive Surgery; Micro-Recon = micro-reconstructive surgery

Criteria	Program-created website (n = 43), n (%)	AAFPRS Handbook (n = 65) n (%)
Program description	38 (88.4)	65 (100)
Research opportunities	24 (55.8)	64 (98.5)
Conference attendance	15 (34.9)	26 (40.0)
General case volume	14 (32.6)	53 (81.5)
Specific case volume	9 (21.4)	20 (30.8)
Mohs	11 (26.2)	50 (76.9)
Aging face	15 (35.7)	58 (89.2)
Rhinoplasty	16 (38.0)	61 (93.8)
Micro-Recon	7 (16.7)	33 (50.8)
Gender-affirming	3 (7.1)	8 (12.3)
Case description	29 (67.4)	63 (96.9)
Didactic learning	9 (20.9)	46 (70.8)
Evaluation criteria	3 (7.0)	0 (0)
Call responsibility	18 (41.9)	62 (95.4)
Teaching responsibility	18 (42.9)	61 (93.9)
Affiliated hospitals	24 (55.8)	59 (90.8)
Program director contact	30 (69.8)	65 (100)
Program coordinator contact	19 (44.2)	0 (0)
Current fellows	19 (44.2)	0 (0)
Current fellow description	10 (23.3)	0 (0)
Graduate fellows	10 (23.3)	0 (0)
Faculty description	18 (41.9)	31 (47.7)
Interview information	3 (7.0)	0 (0)
Business exposure	3 (7.0)	18 (27.7)
Number of positions	12 (29.3)	0 (0)
Benefits	12 (27.9)	64 (98.5)
Vacation/Sick pay	7 (16.3)	34 (52.3)
Salary	10 (23.3)	59 (90.8)
Location information	9 (20.9)	16 (24.6)

**Table 2:** Descriptions of 29 key criteria used for online resource evaluation. Micro-Recon = micro-reconstructive surgery

Criteria	Description
Program description	Provides a general overview of the fellowship program, including its mission and goals
Research opportunities	Outlines the types of research projects or studies fellows can engage in
Conference attendance	Details the provisions or requirements for fellows to participate in academic conferences
General case volume	Provides an estimate of the total number of cases handled throughout the training period
Specific case volume	Specifies the frequency of particular types of procedures
Mohs	Number of Mohs surgeries fellows will likely perform
Aging face	The volume of surgeries focused on aging facial aesthetics
Rhinoplasty	Number of rhinoplasty surgeries fellows will likely perform
Micro-Recon	Number of micro-reconstructive surgeries fellows will likely perform
GenderaAffirming	The volume of surgeries focused on gender-affirming care
Case Description	Provides information about the types and nature of cases fellows will manage
Didactic learning	Describes the educational components of the fellowship, such as structured learning sessions, lectures, and seminars
Evaluation criteria	Outlines the standards used to assess fellows’ performance throughout the fellowship
Call responsibility	Details the on-call duties required of fellows, including the schedule
Teaching responsibility	Describes the roles and expectations for fellows in terms of teaching or mentoring
Affiliated hospitals	Lists the hospitals or medical centers associated with the fellowship
Program director contact	Provides the contact details for the program director
Program coordinator contact	Provides the contact details for the program coordinator
Current fellows	Provides a list of individuals currently enrolled in the fellowship program
Current fellow description	Offers profiles or background information on the current fellows
Graduate fellows	Lists information about alumni from the fellowship program, including their career trajectories post-graduation
Faculty description	Describes the faculty involved in the program, including their expertise
Interview information	Provides details about the interview process
Business exposure	Describes opportunities for fellows to gain insight into the business of medicine, including practice management or marketing strategy
Number of positions	Specifies the number of fellowship positions available annually
Benefits	Details the benefits package for fellows, including health insurance
Vacation/Sick pay	Outlines the policies regarding vacation time and sick leave for fellows
Salary	Provides information on the stipend or salary fellows will receive during the fellowship
Location information	Describes the geographical area or city where the fellowship is based

Among program-created websites, the most frequently reported criteria included program descriptions (38/43, 88.4%), program director contact information (30/43, 69.8%), and case descriptions (29/43, 67.4%). In contrast, the AAFPRS Handbook consistently reported program descriptions (65/65, 100%), program director contact information (65/65, 100%), research opportunities (64/65, 98.5%), and benefits information (64/65, 98.5%).

Notable differences were observed in applicant-facing fellowship characteristics. The AAFPRS Handbook did not report information on current fellows (0/65, 0%), fellowship graduates or post-fellowship placement (0/65, 0%), number of fellowship positions offered annually (0/65, 0%), interview information (0/65, 0%), evaluation criteria (0/65, 0%), or program coordinator contact information (0/65, 0%). In contrast, program-created websites more frequently provided these details, listing current fellows in 19 of 43 programs (44.2%), providing descriptions of current fellows in 10 of 43 programs (23.3%), reporting fellowship graduate information or post-fellowship placement in 10 of 43 programs (23.3%), stating the number of available fellowship positions in 12 of 43 programs (29.3%), offering interview information in 3 of 43 programs (7.0%), and providing program coordinator contact information in 19 of 43 programs (44.2%).

Differences were also evident in the reporting of clinical training content. The AAFPRS Handbook more commonly detailed general case volume (53/65, 81.5%) and procedure-specific case volumes, including Mohs surgery (50/65, 76.9%), aging face procedures (58/65, 89.2%), rhinoplasty (61/65, 93.8%), micro-reconstructive surgery (33/65, 50.8%), and gender-affirming surgery (8/65, 12.3%). Corresponding reporting on program-created websites was lower for these domains, with Mohs surgery reported by 11 of 43 programs (26.2%), aging face procedures by 15 of 43 (35.7%), rhinoplasty by 16 of 43 (38.0%), micro-reconstructive surgery by 7 of 43 (16.7%), and gender-affirming surgery by 3 of 43 programs (7.1%).

Conversely, program-created websites more frequently included information on benefits (12/43, 27.9%), salary (10/43, 23.3%), and vacation or sick leave policies (7/43, 16.3%), whereas these domains were more consistently reported in the AAFPRS Handbook. Information regarding exposure to the business of medicine was limited across both sources, appearing in 18 of 65 handbook entries (27.7%) and only 3 of 43 program-created websites (7.0%). Collectively, these findings demonstrate that the AAFPRS Handbook and program-created websites emphasize different aspects of fellowship training, with the Handbook prioritizing standardized training characteristics and program-created websites more often providing applicant-centered details related to fellowship structure and trainee identity.

University-affiliated versus private practice-based fellowship programs

Among the 65 total FPRS fellowship programs, 53 (81.5%) were university-affiliated, and 12 (18.5%) were private practice-based. University-affiliated programs were more likely to have a program-created fellowship website than private practice-based programs (40/53, 75.5% vs. 3/12, 25.0%) (p < 0.05). Among programs with websites, private practice-based fellowships reported fewer key criteria than academic-affiliated programs, fulfilling an average of 4.7 versus 10.2 of 29 criteria, respectively (p < 0.05). Differences in criteria reporting by program type are summarized in Table 3.

**Table 3:** Descriptive comparison of criteria reported by academic-affiliated versus private practice-based program-created websites. This table displays descriptive reporting of individual criteria by program type. Fisher’s exact test was used to compare the presence of a program-created website by fellowship program type, while Wilcoxon rank-sum testing was performed only for the total number of criteria reported per program and is reported in the text. Micro-Recon = micro-reconstructive surgery

Criteria	Academic-affiliated (n = 40) n (%)	Private practice-based (n = 3) n (%)
Program description	36 (90.0)	2 (66.7)
Research opportunities	24 (60.0)	0 (0.0)
Conference attendance	15 (37.5)	0 (0.0)
General case volume	14 (35.0)	0 (0.0)
Specific case volume	9 (22.5)	0 (0.0)
Mohs	11 (27.5)	0 (0.0)
Aging face	15 (37.5)	0 (0.0)
Rhinoplasty	16 (40.0)	0 (0.0)
Micro-Recon	7 (17.5)	0 (0.0)
Gender-affirming	3 (7.5)	0 (0.0)
Case description	27 (67.5)	2 (66.7)
Didactic learning	9 (22.5)	0 (0)
Evaluation criteria	3 (7.5)	0 (0)
Call responsibility	18 (45.0)	0 (0)
Teaching responsibility	18 (45.0)	0 (0)
Affiliated hospitals	23 (57.3)	1 (33.3)
Program director contact	29 (72.5)	1 (33.3)
Program coordinator contact	19 (47.5)	0 (0)
Current fellows	17 (42.5)	2 (66.7)
Current fellow description	8 (20.0)	2 (66.7)
Graduate fellows	9 (22.5)	1 (33.3)
Faculty description	16 (40.0)	2 (66.7)
Interview information	3 (7.5)	0 (0)
Business exposure	3 (7.5)	0 (0)
Number of positions	11 (27.5)	1 (33.3)
Benefits	12 (30.0)	0 (0)
Vacation/Sick pay	7 (17.5)	0 (0)
Salary	10 (25.0)	0 (0)
Location information	9 (22.5)	0 (0)

Specifically, academic-affiliated programs (n = 40) more consistently reported several core fellowship characteristics than private practice-based programs (n = 3), including program descriptions (36/40, 90.0% vs. 2/3, 66.7%), research opportunities (24/40, 60.0% vs. 0/3, 0.0%), and conference attendance details (15/40, 37.5% vs. 0/3, 0.0%). Academic-affiliated programs also reported procedure-specific case volumes more frequently, including Mohs surgery (11/40, 27.5% vs. 0/3, 0.0%), aging face procedures (15/40, 37.5% vs. 0/3, 0.0%), rhinoplasty (16/40, 40.0% vs. 0/3, 0.0%), micro-reconstructive surgery (7/40, 17.5% vs. 0/3, 0.0%), and gender-affirming procedures (3/40, 7.5% vs. 0/3, 0.0%).

Additional differences were observed in the reporting of program structure and responsibilities. Academic-affiliated programs more frequently included information on call responsibilities (18/40, 45.0% vs. 0/3, 0.0%), teaching responsibilities (18/40, 45.0% vs. 0/3, 0.0%), and affiliated hospitals (23/40, 57.3% vs. 1/3, 33.3%). Program director contact information was also more commonly reported by academic-affiliated programs (29/40, 72.5%) compared with private practice-based programs (1/3, 33.3%). Information regarding benefits (12/40, 30.0%), vacation or sick leave (7/40, 17.5%), and salary (10/40, 25.0%) was reported exclusively by academic-affiliated programs and was not present on private practice-based fellowship websites (0/3, 0.0%).

In contrast, private practice-based programs more frequently reported information about current fellows (2/3, 66.7%) compared with academic-affiliated programs (17/40, 42.5%) and more commonly included faculty descriptions (2/3, 66.7% vs. 16/40, 40.0%).

## Discussion

Online platforms have become essential tools for disseminating information to prospective fellowship applicants. In this study, we evaluated the availability of applicant-relevant information across program-created websites and the AAFPRS Handbook and found that both resources lack important content valued by prospective FPRS fellows. Clear, accessible online communication supports informed decision-making by allowing applicants to evaluate program structure, training opportunities, and alignment with career goals [1,8]. Comprehensive and transparent websites may also enhance recruitment by attracting applicants who are better aligned with a program’s mission and strengths [3-6,8-12].

Overall, program-created websites reported substantially fewer applicant-relevant criteria than the AAFPRS Handbook, reflecting greater variability and inconsistency in publicly available information. While the Handbook more reliably reported standardized training characteristics, it notably omitted applicant-centered details such as current fellows, fellowship graduates, number of positions, interview information, and program coordinator contact information. In contrast, program-created websites more frequently included these applicant-facing elements, though overall reporting remained limited. These findings are consistent with prior analyses across otolaryngology subspecialties, in which fellowship websites similarly failed to meet many applicant-prioritized criteria [3-6].

Importantly, prior survey data indicate that prospective FPRS fellows place a high value on practice type, geographic location, exposure to key surgical techniques, business of medicine training, and the success of recent graduates [2]. However, the criteria most valued by applicants were among the least consistently reported across both online resources, highlighting a disconnect between applicant priorities and publicly available program information. This gap was particularly evident for reporting of specific case volumes, business exposure, and graduate outcomes.

Disparities were most pronounced among private practice-based fellowship programs. Only a minority maintained a dedicated fellowship website, and those that did provided limited content, primarily focusing on general program descriptions and fellow identity. The absence of detailed reporting on case mix and practice management may reflect differing recruitment strategies in private practice settings, where marketing efforts often prioritize patient-facing aesthetics over educational transparency [13-16]. Nevertheless, this lack of standardized, accessible information may place applicants interested in private practice training at a disadvantage and increase reliance on informal networks and word-of-mouth communication.

Overall, this study demonstrates that the AAFPRS Handbook and program-created websites offer complementary but incomplete information for prospective FPRS applicants. The Handbook serves as a more consistent and comprehensive foundation for data gathering. In contrast, program-created websites may be more informative for applicants seeking insight into fellow identity and career trajectories. Greater standardization and improved depth of reporting, particularly among private practice-based programs, could reduce applicant reliance on informal information channels and improve equity in fellowship program evaluation.

This study has several limitations. The evaluated criteria, while derived from prior literature and applicant surveys, have not been formally validated. The small number of private practice-based programs with websites limited statistical power for subgroup analyses. Additionally, the cross-sectional design does not capture changes to online content over time. Finally, we were unable to assess how website content influences applicant perceptions or program desirability, which would require direct applicant surveys. Future studies should incorporate applicant perspectives, assess longitudinal changes in online reporting, and explore the role of social media platforms in shaping fellowship recruitment and decision-making.

## Conclusions

This study highlights that both program-created FPRS fellowship websites and the AAFPRS Handbook incompletely report information that applicants consider most important, limiting fellows’ ability to meaningfully assess program fit. While the AAFPRS Handbook provides more consistent coverage across many domains, it omits applicant-centered details such as current fellows, graduate outcomes, and interview logistics. Program-created websites, in contrast, are highly variable and frequently absent, with particularly pronounced deficiencies among private practice-based fellowships. Compared with academic programs, private practice fellowships are far less likely to have dedicated websites and report substantially fewer criteria, especially regarding surgical case mix, research exposure, and practice management, despite these factors being highly valued by applicants. Addressing these disparities through more comprehensive and standardized online reporting, especially among private practice programs, may improve transparency, applicant decision-making, and overall fellowship alignment in FPRS.
